# *De Novo* Sequencing-Based Transcriptome and Digital Gene Expression Analysis Reveals Insecticide Resistance-Relevant Genes in *Propylaea japonica* (Thunberg) (Coleoptea: Coccinellidae)

**DOI:** 10.1371/journal.pone.0100946

**Published:** 2014-06-24

**Authors:** Liang-De Tang, Xing-Min Wang, Feng-Liang Jin, Bao-Li Qiu, Jian-Hui Wu, Shun-Xiang Ren

**Affiliations:** 1 Engineering Research Center of Biological Control, Ministry of Education, College of Natural Resource and Environment, South China Agricultural University, Guangzhou, China; 2 Environment and Plant Protection Institute, Chinese Academy of Tropical Agriculture, Haikou, China; Institute of Vegetables and Flowers, Chinese Academy of Agricultural Science, China

## Abstract

The ladybird *Propylaea japonica* (Thunberg) is one of most important natural enemies of aphids in China. This species is threatened by the extensive use of insecticides but genomics-based information on the molecular mechanisms underlying insecticide resistance is limited. Hence, we analyzed the transcriptome and expression profile data of *P. japonica* in order to gain a deeper understanding of insecticide resistance in ladybirds. We performed *de novo* assembly of a transcriptome using Illumina's Solexa sequencing technology and short reads. A total of 27,243,552 reads were generated. These were assembled into 81,458 contigs and 33,647 unigenes (6,862 clusters and 26,785 singletons). Of the unigenes, 23,965 (71.22%) have putative homologues in the non-redundant (nr) protein database from NCBI, using BLASTX, with a cut-off E-value of 10^−5^. We examined COG, GO and KEGG annotations to better understand the functions of these unigenes. Digital gene expression (DGE) libraries showed differences in gene expression profiles between two insecticide resistant strains. When compared with an insecticide susceptible profile, a total of 4,692 genes were significantly up- or down- regulated in a moderately resistant strain. Among these genes, 125 putative insecticide resistance genes were identified. To confirm the DGE results, 16 selected genes were validated using quantitative real time PCR (qRT-PCR). This study is the first to report genetic information on *P. japonica* and has greatly enriched the sequence data for ladybirds. The large number of gene sequences produced from the transcriptome and DGE sequencing will greatly improve our understanding of this important insect, at the molecular level, and could contribute to the in-depth research into insecticide resistance mechanisms.

## Introduction

The Coccinellidae family consists of almost 6000 species that are distributed worldwide. The beetle species of this family have been used successfully as biological control agents in crop protection. The ladybird beetle, *Propylaea japonica* (Coleoptera: Coccinellidae), is a common and abundant indigenous natural enemy of aphids, whitefly species, scale insects and small caterpillars in China [1]–[3]. It has four to five generations per year in northern China and seven to eight generations per year in southern China. Both larval and adult life stages are predaceous, with a very high rate of daily prey consumption. Furthermore, *P. japonica* can reproduce under poor diet conditions and survive a wide range of temperatures without undergoing estivation [4]. For these reasons, it is considered to be one of the best biocontrol agents in the field [5], [6]. Biological control is usually accompanied by chemical control for practical applications in the field. As the insect pests and their natural enemies survive in the same habitat, the influence of insecticides on natural predators should not be ignored in integrated pest management (IPM). Imidacloprid, as a selective insecticide, appears to be specific to Homopterans (e.g. aphids, whiteflies and planthoppers), with high systemic activity [7]–[9], but is also highly toxic to a few species of Coleoptera (beetles) [10]. The predator *P. japonica* is one of the main prey species of the target pests of imidacloprid and is therefore becoming more and more threatened by this insecticide. To date, the molecular mechanisms of the ladybird response to insecticides are not clearly understood. Most published data is focused on the side-effects and biological characterization effected by insecticides [11]–[16].

Despite being by far the most species-rich order of insects, Coleoptera is under-represented in the available arthropod sequence information. However, a transcriptome resource has recently been developed for insecticide resistance mechanisms in *Cryptolaemus montrouzieri* Mulsant [17]. The development of a comprehensive resource for sequence information in Coccinellid beetles is essential to better understand the biology of these ecologically and economically important species. It may also help to improve their use as biological agents for controlling insect pests. Research on the transcriptome is essential for deciphering the functional elements of the genome and understanding their expression and regulation. The recently developed Solexa/Illumina RNA-seq and digital gene expression (DGE) based next-generation sequencing (NGS) technologies have dramatically changed the way that resistance-relevant genes in insects are identified. Based on NGS technologies, we generated more than 27 million raw reads for *de novo* assembly and annotation, without a genome reference sequence. A comprehensive analysis of the global response to insecticide stress in the ladybird can aid in the in-depth investigation of candidate resistance-relevant genes. These data provide a rich resource that will be useful for the screening and analysis of ladybird functional genes and also provide a novel method for understanding the molecular mechanisms that underlie resistance in non-model species.

## Materials and Methods

### Insect samples

The laboratory colony of *P. japonica* was originally collected from a vegetable field in the Mengshan Mountain region (Shandong province, China) during June, 2010. It was maintained for more than two years on *Aphis craccivora* Koch, at 26±1°C, 60–80% relative humidity (RH) and a 14:10 (L:D) photoperiod, in climate chambers at the Engineering Research Center of Biological Control, Ministry of Education, China. During this period the colony had no contact with insecticides. These beetles were considered to be a susceptible strain (designated SUS) and used for transcriptome sequencing, as described. The following developmental stages were collected: eggs within 24 h after oviposition (egg); first-instar larvae (1^st^); second-instar larvae (2^nd^); third-instar larvae (3^rd^); fourth-instar larvae (4^th^); pre-pupae (pre-p); pupae (p); and newly-emerged adults, in a 1∶1 female/male ratio (adu). For DGE sequencing, two samples with each four adults were collected. (1) A moderate imidacloprid-resistant (35.1 fold) adult-strain sample (designated R-mid). Resistance was selected in the laboratory using a standard glass tube residual film bioassay. Selection was carried out at every generation, while the resistance analysis was investigated at intervals of every two generations. The selection concentration was increased in accordance with the LC_50_ value of bioassay results of the parent generation. Mortality was maintained at approximately 60–80%. Surviving adults were transferred out of insecticide conditions and the populations were allowed to regenerate. At least 230 adults were selected for each cycle. (2) In parallel, the SUS adults were used as the negative control samples. Each sample was immediately frozen in liquid nitrogen after collection.

### RNA preparation for RNA-Seq

Total RNA was isolated using the UNlQ-10 Column Trizol Total RNA Isolation Kit (Sangon), in accordance with the manufacturer's instructions. The integrity of the RNA was confirmed using a 2100 Bioanalyzer (Agilent Technologies), with a minimum RNA integrity number (RIN) of 7.0. Magnetic beads labelled with Oligo (dT) were used to purify mRNA from the total RNA (a mixture of RNA from different developmental stages, mixed at an equal ratio). The RNA was mixed with fragmentation buffer to create short fragments. These were used as templates for first-strand cDNA synthesis with random hexamer primers. Second-strand cDNA was synthesized using a reaction system of buffer, dNTPs, RNaseH and DNA polymerase I. Short fragments were purified with the QiaQuick PCR extraction kit and resolved with EB buffer for end reparation and poly (A) addition. These were then connected with adapters and suitable fragments were selected as templates for PCR amplification, to create the final cDNA library. After qualification of the sample library was performed using the ABI StepOnePlus Real-Time PCR System, the library was sequenced using the Illumina HiSeq 2000 kit, in accordance with the manufacturer's recommendations.

### Bioinformatics analysis of the transcriptome

Image data and quality value calculations were performed using Illumina GA Pipeline v1.6. The raw reads were filtered by removing adaptor reads, unknown nucleotide (>5%) reads and low quality reads. The clean reads were used for transcriptome *de novo* assembly, using the short-reads assembling program, Trinity [18]. This software partitions the sequence data into many individual de Bruijn graphs, each representing the transcriptional complexity at a given gene or locus. Each graph is independently processed to extract full length splicing isoforms and to tease apart transcripts derived from paralogous genes. The resulting sequences from Trinity, without Ns and that could not be extended at either end, were defined as unigenes. The unigenes were subjected to BLAST searches and annotation against the NR protein database, the Swiss-Prot database, the KEGG database and the COG database, with a cut-off E-value of 10^−5^. Sequence directions were determined in accordance with the best hits in the database. When different databases gave conflicting results, a priority order of NR, Swiss-Prot, KEGG and COG was followed. When a unigene did not align to any of the above databases, ESTScan [19] was used to predict its coding regions and to determine its sequence direction (5′→3′). Simple sequence repeats (SSRs) were identified using MicroSAtellite software, whilst single nucleotide polymorphisms (SNPs) were predicted using SOAPsnp software.

### Analysis of genes related to insecticide detoxification and target enzymes

Sequences of genes related to insecticide resistance, such as detoxification enzymes (cytochrome P450s, glutathione S-transferases and carboxylesterase) and insecticide targets (acetylcholinesterase, gamma-aminobutyric acid, nicotinic acetylcholine receptor subunits and sodium channel), were identified using the BLAST nr database, with an E-value cut-off of 1E^−5^. All identified sequences that returned a BLAST hit or showed high homology, as determined by alignment results, were eliminated as allelic variants or different regions of the same gene.

### DGE library preparation and sequencing

The RNA was extracted separately from two adult samples: a moderately imidacloprid-resistant sample and the SUS adult as the negative control group, using the UNlQ-10 Column Trizol Total RNA Isolation Kit (Sangon), in accordance with the manufacturer's instructions. Approximately 10 µg of RNA from each sample were used to construct the DGE libraries. The mRNA was treated as described in cDNA library construction. The fragments were purified by agarose gel electrophoresis and enriched by PCR amplification. The library products were then ready for sequencing via Illumina HiSeq™ 2000, using paired-end technology in a single run.

### Mapping DGE tags to transcriptome databases

Base calling was used to convert the original image data into sequences. All raw sequence reads were filtered using the Illumina pipeline before mapping reads to the reference transcriptome databases. Low quality reads, such as those with adaptor sequences, those in which the percentage of unknown bases (N) was more than 10% and reads in which more than 50% of bases had a quality value ≤5, were removed from data analysis. Clean reads were mapped to reference sequences (transcriptome databases) using SOAPaligner/SOAP2 [20]. No more than two mismatches were allowed in the alignment. The gene expression level was calculated using the RPKM method (reads per kb per million reads) [21]. If there was more than one transcript for a given gene, the longest was used to calculate its expression level and coverage. In further analyses, the differentially expressed tags were used for mapping and annotation.

### Screening of differentially expressed genes

To identify genes that showed differential expression between the two samples, the false discovery rate (FDR) method described by Audic *et al.* [22] was used to determine the threshold of the P-value in multiple tests and analyses. We used “FDR≤0.001 and the absolute value of log2Ratio≥1” as the threshold to judge the significance of difference in gene expression. More stringent criteria, with smaller FDR and bigger fold-change values, can be used to identify differentially expressed genes. Genes with similar expression patterns usually show a functional correlation. We performed a cluster analysis of gene expression patterns with cluster software [23] and Java Treeview [24] software. The genes that were expressed at different levels across the samples were further annotated by the Gene Ontology (GO) function analysis, using the hypergeometric test and KEGG pathway analysis.

### Experimental validation

To confirm the DGE results, we designed 16 pairs of primers to perform quantitative PCR (qPCR) analysis on 13 up-regulated genes and three down-regulated genes. The primers are shown in Table S1. The RNA samples used for the qPCR assays were those used for the DGE experiments and we also isolated RNA independently from biological replicates. The first cDNA strand was synthesized from 1.0 µg of total RNA using the PrimeScript RT reagent Kit (TaKaRa). The qPCR was performed using a BIO-Rad IQ5 Real-Time PCR system (Bio-Rad) with SYBR-Green detection (SYBR Premix Ex Taq, TaKaRa), in accordance with the manufacturer's instructions. Each reaction was run in triplicate, after which the average threshold cycle (Ct) was calculated per sample. The β-actin gene was used to normalize expression levels and the relative expression of genes was calculated using the 2^−ΔΔCt^ method.

## Results

### RNA-Seq and sequence assembly

A library of all life stages (eggs, first-instar larvae, second-instar larvae, third-instar larvae, pupae and adults) of *P. japonica* was constructed by Illumina sequencing in a single run, which generated 27,243,552 reads (submitted to NCBI Short Read Archive (SRA) database, Accession No. SRP042242) in total, and 2,311,855,380 nucleotides (nt). These were assembled into 81,458 contigs and 33,647 unigenes (6,862 clusters and 26,785 singletons) (Table 1). The length of the clusters varied from 60 to 4,615 base pairs (bp), with an average contig length of 759 bp and a total of 2,962,366 bp. The singletons ranged from 50 to 863 bp, with an average length of 313 bp and a total of 9,919,703 bp (Figure S1). These non-redundant transcripts were prepared as a transcriptome database to search for insecticide-resistance relevant genes by DGE.

**Table 1:** Summary of the transcriptome.

Total raw reads	27,243,552
Total clean reads	25,678,282
Total clean nucleotides (nt)	2,311,855,380
Total number of contigs	81,458
Total number of unigenes	33,647

### Annotation of predicted proteins

**Figure 1:**
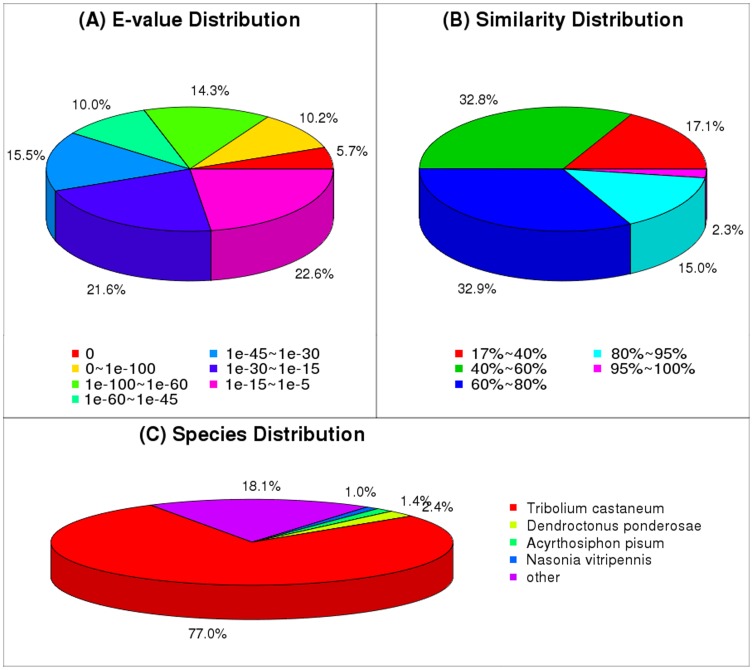
Species distribution of the BLASTX matches of the transcriptome unigenes. This figure shows the species distribution of unigene BLASTX matches against the nr protein database (cutoff value E<10^−5^) and the proportions for each species.

Unigene sequences were annotated by searching the non-redundant (nr) NCBI protein database using BLASTX, with a cut-off E-value of 10^−5^. A total of 23,965 distinct sequences (71.22% of unigenes) matched known genes. The majority of sequences (77.0%) had strong homology with *Tribolium castaneum* (Figure 1). Of these, 2.4% of the unigenes were best matched to sequences from *Dendroctonus ponderosae*, followed by *Acyrthosiphon pisum* (1.4%), *Nasonia vitripennis* (1.0%), and 18.1% from other species.

### Unigene function annotation

**Figure 2:**
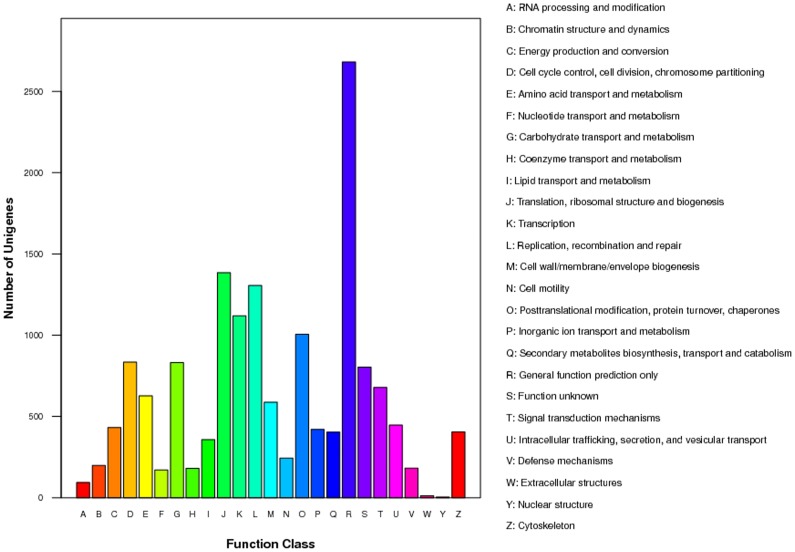
Classification of the clusters of orthologous groups (COG) for the transcriptome of *Propylaea japonica*. Unigenes (15,405) were annotated and divided into 25 specific categories.

To analyze putative protein functions, the database of clusters of orthologous groups (COG) was used to predict and classify possible functions of the unigenes. Using sequence homology, 15,405 unigenes were annotated and divided into 25 specific categories (Figure 2). Among these categories, the cluster for ‘General function prediction’, which contained 2,680 unigenes (17.40%), represented the largest group, followed by ‘Translation, ribosomal structure and biogenesis’ (1,384, 8.98%), ‘Replication, recombination and repair’ (1,305, 8.47%), ‘Transcription’ (1,119, 7.26%), and ‘Post-translational modification, protein turnover and chaperones’ (1,005, 6.52%). The categories ‘Nuclear structure’ (4, 0.03%) and ‘Extracellular structures’ (12, 0.08%) represented the smallest groups.

**Figure 3:**
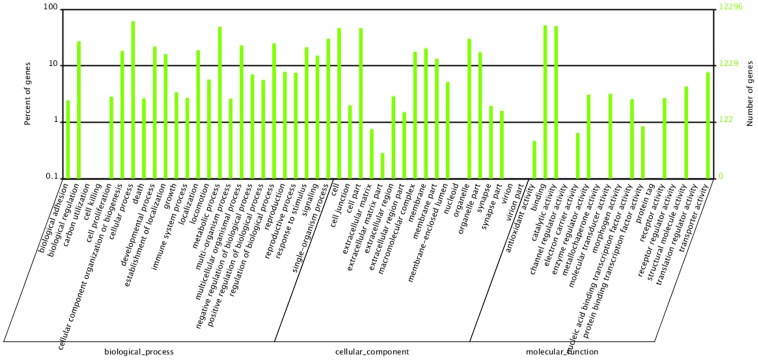
Classification of the gene ontology (GO) for the transcriptome of *Propylaea japonica*. Transcripts (12,296) were categorized into 60 function groups. The right y-axis indicates the number of genes in a category, whereas the left y-axis indicates the percentage of a specific category of genes in that main category.

In a GO analysis, a total of 12,296 transcripts, out of 23,965 Nr-annotated transcripts, were categorized into 60 function groups in each of the three ontologies: biological processes, cellular component and molecular function (Figure 3). We observed a large number of unigenes in the categories of ‘Cellular process’ (7,570), ‘Binding’ (6,404), ‘Metabolic process’ (6,012), ‘Catalytic activity’ (6,220) and ‘Cell part’ (5,671), whilst fewer than 10 unigenes were observed in ‘Translation regulator activity’ (9), ‘Nucleoid’ (9), ‘Morphogen activity’ (8), ‘Carbon utilization’ (5), ‘Cell killing’ (4), ‘Virion’ (4), ‘Virion part’ (4), ‘Metallochaperone activity’ (4), ‘Channel regulator activity’ (3), ‘Receptor regulator activity’ (3) and ‘Protein tag’ (1) (Figure 3).

### Unigene metabolic pathway analysis

The unigene metabolic pathway analysis was conducted using the Kyoto Encyclopedia of Genes and Genomes (KEGG) annotation system. We mapped 16,147 unigenes to 255 KEGG pathways (Table S2). The most enriched pathways included metabolic pathways (2,276, 14.1%), RNA transport (548, 3.39%), regulation of actin cytoskeleton (530, 3.28%), focal adhesion (516, 3.20%), pathways in cancer (512, 3.17%), Huntington's disease (482, 2.99%), and spliceosome (468, 2.90%). These annotations provide a valuable resource for the investigation of specific processes, functions and pathways in the ladybird.

### Transcripts that encode specific genes associated with insecticide resistance

To obtain unique sequences related to insecticide resistance, non-redundant transcripts associated with insecticide targets and metabolism were analyzed (Table 2). As showed in Table 2, we observed that many transcripts of complex multi-gene enzyme systems were involved in resistance. These systems included esterases, such as carboxylesterase, amidohydrolase, phosphodiesterase, cytochrome P450s and glutathione S-transferases. A number of transcripts that encode insecticide targets, such as acetylcholinesterase (AChE), nicotinic acetylcholine receptor subunits (nAChRs), the GABA-gated ion channel and the sodium channel, were also found. Other enzymes that may be related to the regulation of insecticide resistance were also analyzed (Table 2).

**Table 2:** Genes related to insecticide targets and metabolism.

Insecticide metabolism and targets	CD-containing transcripts number
Mixed-function oxidases	
Cytochrome P450	129
Glutathione S-transferase	
Glutathione S-transferase	23
Esterases	
Carboxylesterase	59
Amidohydrolase	1
Phosphodiesterase	22
Ligand-gated ion channels	
Nicotinic acetylcholine receptor	14
GABA-gated ion channel	7
Glutamate receptor	11
voltage-gated ion channels	
Sodium channel	1
Voltage-gated ion channel	9
Other enzymes	
Catalase	2
NADH dehydrogenase	22
Trypsin	21
Superoxide dismutase	11
Acetylcholinesterase	10

### Putative molecular markers

A total of 128,893 putative SNPs (85,056 transitions and 43,837 transversions) were predicted using SOAPsnp software (Li, 2009) (Table S3). In addition, 356 SSRs were identified, of which 71% were tri-nucleotide repeats, 11% were di-nucleotide repeats, 13% were mono-nucleotide repeats, 1.7% were quad-nucleotide repeats, 1.4% were penta-nucleotide repeats and 1.4% were hexa-nucleotide repeats (Table S4). Identification of such molecular markers could provide a platform to improve the understanding of the biology and ecology of this ladybird.

### Digital gene expression (DGE) library sequencing

Digital gene expression analysis was performed to obtain a global view of the ladybird transcriptome under insecticide stress. Two DGE libraries were constructed to identify gene expression profiles of the different imidacloprid-resistant levels, which were the susceptible strain and moderately resistant strain. A total of 6.32 and 6.99 million raw reads were generated from the above two strains, respectively, using massively parallel sequencing on the IIlumina platform. After removal of low-quality reads, the total number of clean reads per library was 6.22 and 6.71 million, for the susceptible and moderately resistant strains, respectively (Figure S2). Of these clean reads, 4.91 (78.80%) and 5.52 (82.27%) million were mapped to unigenes in the susceptible and moderately resistant strain libraries, respectively (Table 3). The high percentage of clean reads (95.93% and 98.52%) reflected the high quality of sequencing (Figure S3). More than 25% of genes showed 90–100% coverage in each strain. Approximately 3% of the genes showed 0–10% coverage. The distribution of coverage of genes in the different strains is shown in Figure S3.

**Table 3:** Alignment statistics of the RNA-Seq analysis.

Summary	S-SuS	R-mid
Total reads	6,710,697 (100.00%)	6,224,800 (100.00%)
Total base pairs	328,824,153 (100.00%)	305,015,200 (100.00%)
Total mapped reads	5,520,945 (82.27%)	4,905,375 (78.80%)
Perfect match	3,975,169 (59.24%)	3,148,053 (50.57%)
< = 2 bp mismatch	1,545,776 (23.03%)	1,757,322 (28.23%)
Unique match	4,660,267 (69.45%)	4,233,287 (68.01%)
Multi-position match	860,678 (12.83%)	672,088 (10.80%)
Total unmapped reads	1,189,752 (17.73%)	1,319,425 (21.20%)

### Gene expression profile analysis of responses to insecticide

**Figure 4:**
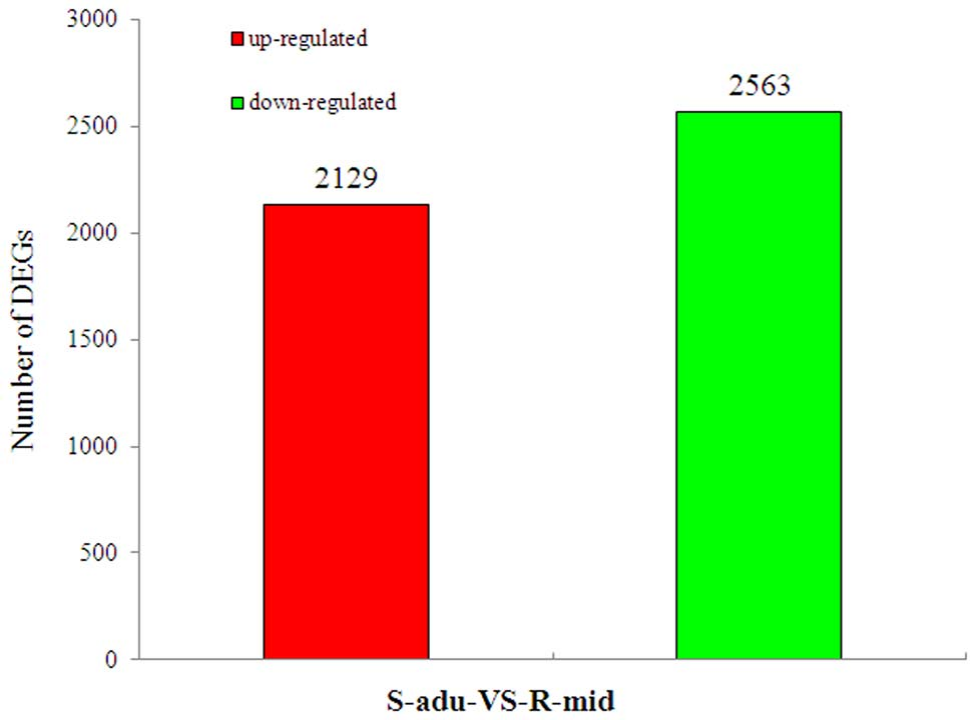
Summary of differentially expressed genes in pairwise comparisons between S-adu and R-mid.

To compare differentially expressed (DE) genes between the libraries (R-mid/SUS), the level of gene expression was determined by normalizing the number of unambiguous tags in each library to reads per kb per million reads (RPKM). To determine whether insecticide resistance results in significant changes in gene expression, differential DGE analysis was performed using a strict Bayesian algorithm [22]. Genes that were found to have significant differences in expression were identified between the pairwise comparisons of the susceptible strain vs. moderately resistant strain (SUS vs. R-mid) (Figure 4, Table S5,). When compared with the susceptible strain, a total of 4,692 genes were affected in the moderately resistant strain; 2,129 genes were up-regulated and 2,563 were down-regulated.

**Figure 5:**
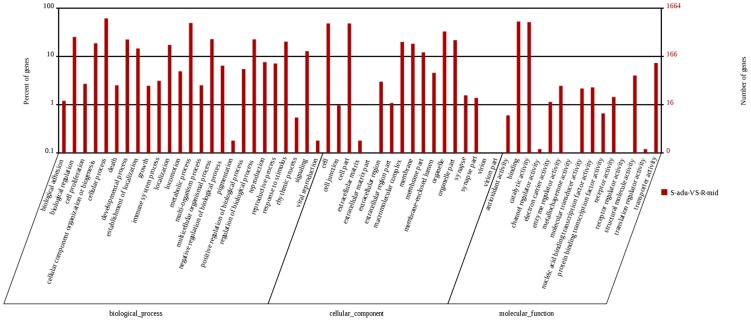
Classification of the gene ontology (GO) for the DGE of *Propylaea japonica*.

Using the GO classification, differentially expressed genes were characterized into three groups: biological process, cellular component and molecular function (Figure 5). Amongst the biological process assignments, a high percentage of genes were concentrated in the cellular process category (1,023 genes, 21.8%), predominantly in the microtubule-based movement, spindle organization, and metabolic process category (821, 17.5%). Glycolysis and mitochondrial ATP synthesis coupled electron transport were also enriched with differentially expressed genes that fell into this category (Figure S4). The cellular component group showed a significant percentage of genes assigned to cell part (802 genes, 17.5%) and organelle (548 genes, 11.7%) categories, where they were mainly concentrated in the microtubule dynein complex and mitochondrial respiratory chain (Figure S5). Most of the genes that fell into the molecular function group (Figure S6) were involved in binding (879 genes, 18.7%), followed by catalytic activity (855 genes, 18.2%), where they enriched oxidoreductase activity, microtubule motor activity and hydrogen ion transmembrane transporter activity categories.

To find more genes that are responsive to insecticides, we selected the imidacloprid-resistant ladybird group. Stress by imidacloprid treatment resulted in large alterations in the ladybird transcriptome profile, such as significant up- or down-regulation of 4,692 transcripts. Amongst the affected genes, 125 known genes (Table S6) were found to encode detoxification enzymes and insecticide targets. These are involved in insecticide response KEGG pathways, such as metabolic pathways, drug metabolism cytochrome P450, retinol metabolism, linoleic acid metabolism, glutathione metabolism, arachidonic acid metabolism, metabolism of xenobiotics by cytochrome P450, complement and coagulation cascades, insect hormone biosynthesis, steroid hormone biosynthesis, steroid biosynthesis, drug metabolism enzymes, tyrosine metabolism, oxidative phosphorylation, neuroactive ligand-receptor interaction, glutamatergic synapse, protein digestion and absorption, cytokine-cytokine receptor interaction, ubiquitin mediated proteolysis, chemokine signaling pathway and purine metabolism.

In our DGE libraries, we also found that numerous genes associated with material and energy catabolism were over-transcribed. These included heat shock proteins (20 genes), ATP synthase (15 gene), ATP-binding cassette transporters (four genes) and some Bt receptor genes that are indirectly related to resistance, such as aminopeptidase N (three genes), glycosyltransferase (two genes) and cadherin (one gene) (Table S7).

### Experimental validation

**Figure 6:**
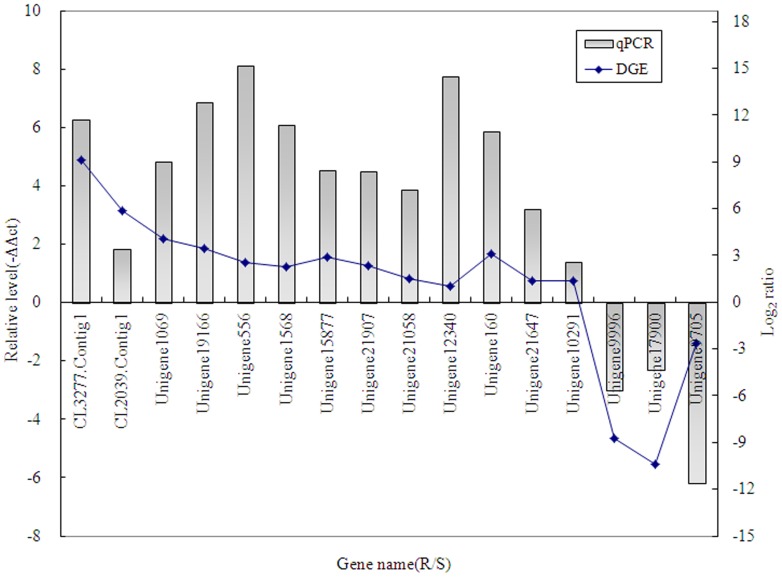
qRT-PCR validation of DGE results. Thirteen up-regulated genes and three down-regulated genes have been identified by qRT-PCR. The 13 up-regulated genes include eight cytochrome P450 genes (CL3277.Contig1, CL2039.Contig1, Unigene1069, Unigene19166, Unigene556, Unigene1568, Unigene15877, Unigene21907), two glutathione S-transferase genes (Unigene21058, Unigene12340), two esterases (Unigene160, Unigene21647) and one acetylcholine receptor (Unigene10291). The three down-regulated genes are cytochrome P450 (Unigene9996), cuticular protein (Unigene17900) and nAChR (Unigene9705). The left y-axis indicates the relative expression level, by qRT-PCR, and the right y-axis indicates the log_2_Ratio of R-mid/SUS by DGE.

To confirm the quality of the transcriptome data and the differential DGE results by Solexa/Illumina sequencing, we selected 13 up-regulated genes (eight cytochrome P450 (CL3277.Contig1, CL2039.Contig1, Unigene1069, Unigene19166, Unigene556, Unigene1568, Unigene15877 and Unigene21907), two glutathione S-transferases (Unigene21058, Unigene12340), two esterases (Unigene160, Unigene21647), one acetylcholine receptor (Unigene10291)) and three down-regulated genes (cytochrome P450 (Unigene9996), cuticular protein (Unigene17900) and nAChR (Unigene9705)) for qRT-PCR confirmation. The values for the resistant samples (R-mid) are presented as the fold change in gene expression, normalized to the β-actin gene, relative to the SUS samples. The qPCR analysis confirmed the direction of change in gene expression detected by DGE analysis (Figure 6).

## Discussion

The ladybird beetle is one of the biggest insect groups and the majority of its species are predacious enemies of insect pests. Most studies of this insect group have focused on biology and ecology. However, a lack of genetic information is still a barrier to our knowledge of this beetle group. The advent of next-generation sequencing (NGS), which has been developed for rapid sequencing and transcriptomic research, has allowed us to use RNA-seq and DGE methods to analyze the *P. japonica* transcriptome and insecticide resistance mechanisms. We generated more than 27 million raw reads with Illumina sequencing. With *de novo* assembly, the raw reads were further assembled into 33,647 unigenes using the methods in the Velvet and Oases programs. For the ladybird, as a non-model organism with no reference genome sequence, the assembly by Velvet, followed by the Oases program, was an improvement on the use of Velvet alone. It was also better than other programs based on assessment parameters such as N50 length, average contig length and sequence similarity with closely related species [17], [25]–[26]. Amongst the high quality unigenes, the BLASTX analysis of GenBank showed that 23,964 (71.22%) have significant homology to functional genes that encode specific proteins. As expected, the red flour beetle, *T. castaneum*, is the species that returned the most BLAST hits (77.0%) against the *P. japonica* transcripts. We also found a significant percentage of transcripts (18.1%) to be unique to *P. japonica*, which could perhaps be attributed to the presence of novel genes. However, this result could also be explained by the fact that *T. castaneum* is the only beetle species with a completely sequenced genome [27]. Although a recent study on the first ladybird transcriptome resource of *C. montrouzieri* is available [17], no further information can be found in the NCBI database. The new transcriptome data reported here complements the scarce ladybird sequence resources in GenBank.

Food safety has been receiving more attention than ever before in modern society. This suggests that natural enemies, as a means of biological control, should be given more attention by plant protection programmers. Biological control is usually accompanied by chemical control for practical applications in the field, which means that natural enemies are often threatened by the use of insecticides. Molecular resistance mechanisms are still unknown and the main obstacle to further research is the lack of genetic information. In order to assist studies on insecticide resistance, we surveyed our transcriptome database and insecticide resistance DGE libraries to identify the most important enzymes related to the metabolism of insecticides and genes that encode the protein targets.

Cytochrome P450s (P450s), glutathione S-transferases (GSTs) and esterases (ESTs) are recognized as three major detoxifying enzymes that are involved in detoxification of different chemical classes [28]–[32]. Acetylcholinesterase (AChE) and nicotinic acetylcholine receptor subunits (nAChRs) are the main genes that encode insecticide targets. During the recent targeting of a Coccinellidae species (*C. montrouzieri*), with next generation sequencing technologies, genes related to insecticide resistance, such as P450, GSTs, CarEs and nAChRs, were identified [17]. However, no information on any of these genes has been reported for *P. japonica*. In our transcriptome database, 129 transcripts that encode P450 genes were found, 23 unique GST sequences were selected and 82 esterase gene sequences were discovered (Table 2). We also identified 10 AChE and 14 nAChRs unigenes in our transcriptome database (Table 2). In this way, our work provides a basis to help understand the mechanisms of insecticide resistance and could greatly improve future studies of this natural enemy at the molecular level.

To find further insecticide resistance related genes, we constructed DGE libraries for two strains with different imidacloprid resistance levels. By targeting a defined cDNA library, the DGE method can generate broader transcriptome coverage and a higher number of cDNA tags per gene, which can lead to more precise gene transcription data. Provided that a reference genome or transcriptome database is available and the aim is to quantify transcript levels between different biological samples, this method is perfectly suited for deep transcriptome analysis [33]. We obtained 5,520,945 (82.27%) and 4,905,375 (78.80%) tag-mapped reads from the SUS and R-mid strains, respectively, by mapping the tags to a transcriptome reference database (Table 3). Using the Solexa/Illumina DGE method, a total of 4,692 genes exhibited differential expression in the moderately resistant strain, when compared with the susceptible strain. Of these genes, we found 125 known genes that encode detoxification enzymes and insecticide targets (Table S6), and 43 putative genes that are involved in insecticide response (Table S7). However, genes that are related to insecticide resistance represent only a small proportion of the total transcripts that were found in our transcriptome database. This agrees with a recent study that showed a similar transcriptome response to this insecticide [17]. Identification of differentially expressed genes under insecticide stress is useful for further analysis of the mechanism of insecticide resistance in insects and may identify candidate genes for enhancement of this characteristic.

In conclusion, this study is the first to report genetic information on *P. japonica* from sequenced transcriptome and constructed DGE libraries. These efforts revealed a large number of genes, of both known and unknown functions, which have greatly enriched sequence information of the ladybird. We have also identified genes that are potential candidates for conferring insecticide resistance in *P. japonica*. These include genes that encode enzymes putatively involved in metabolic detoxification of xenobiotics and those that encode the target proteins of the major chemical classes of insecticides. With these important genetic resources, we plan to further validate the gene functions that are associated with insecticide resistance in *P. japonica* using RNA interference (RNAi) technology.

## Supporting Information

Figure S1 **Summary of** ***Propylaea japonica*** **transcriptome sequences.** (A) Length distribution of contigs, (B) Length distribution of unigenes.
(TIF)

Figure S2 **The quality of** ***Propylaea japonica*** **DGE sequences.** (A) SUS, (B) R-mid.
(TIF)

Figure S3 **The distribution of gene coverage of** ***Propylaea japonica*** **DGE sequences.** (A) SUS, (B) R-mid.
(TIF)

Figure S4 **Enrichment analysis of the biological process group of gene ontology (GO) for the DGE of** ***Propylaea japonica*** **.**
(TIF)

Figure S5 **Enrichment analysis of the cellular component group of gene ontology (GO) for the DGE of** ***Propylaea japonica*** **.**
(TIF)

Figure S6 **Enrichment analysis of the molecular function group of gene ontology (GO) for the DGE of** ***Propylaea japonica*** **.**
(TIF)

Table S1 **The primer of qPCR.**
(DOC)

Table S2 **The unigene metabolic pathway analysis of** ***Propylaea japonica*** **was conducted using the Kyoto Encyclopedia of Genes and Genomes (KEGG) annotation system.**
(DOC)

Table S3 **Predicted single nucleotide polymorphisms (SNP) in** ***Propylaea japonica*** **sequences.**
(DOC)

Table S4 **Summary of microsatellite loci predicted in** ***Propylaea japonica*** **sequences.**
(DOC)

Table S5 **The differentially expressed genes (DEGs) between SUS and R-mid strain.**
(XLS)

Table S6 **Representatives of putative insecticide resistant genes as predicted by DGE.** Limitations of all significantly different expressed genes between R-low (or R-mid) and SUS are based on FDR≤0.001 and the absolute value of log2Ratio≥1. The log2Ratio(R-mid/SUS) indicates the change of gene expression; a positive number means up-regulation and a negative one means down-regulation.
(DOC)

Table S7 **Representatives of putative indirectly related to insecticide resistant genes as predicted by DGE.** Limitations of all significantly different expressed genes between R-mid and SUS are based on FDR≤0.001 and the absolute value of log2Ratio≥1. The log2Ratio(R-mid/SUS) indicates the change of gene expression; a positive number means up-regulation and a negative one means down-regulation.
(DOC)
