# Supplementary figures and images for: De Novo Sequencing-Based Transcriptome and Digital Gene Expression Analysis Reveals Insecticide Resistance-Relevant Genes in Propylaea japonica (Thunberg) (Coleoptea: Coccinellidae)
Source: PLoS One. 2014 Jun 24;9(6):e100946. doi: 10.1371/journal.pone.0100946 (PMC4069172; doi:10.1371/journal.pone.0100946)

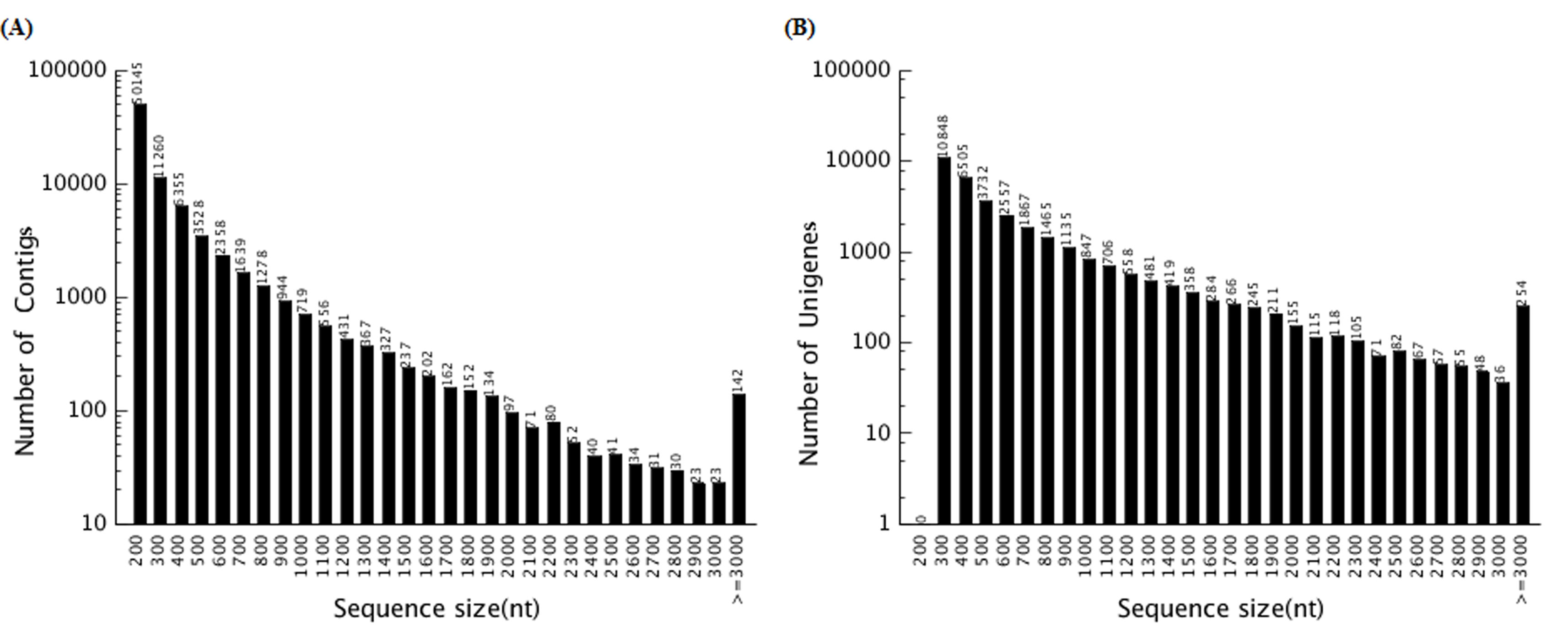

Supplement: Figure S1 — Summary of Propylaea japonica transcriptome sequences. (A) Length distribution of contigs, (B) Length distribution of unigenes. (TIF) [file pone.0100946.s001.tif]

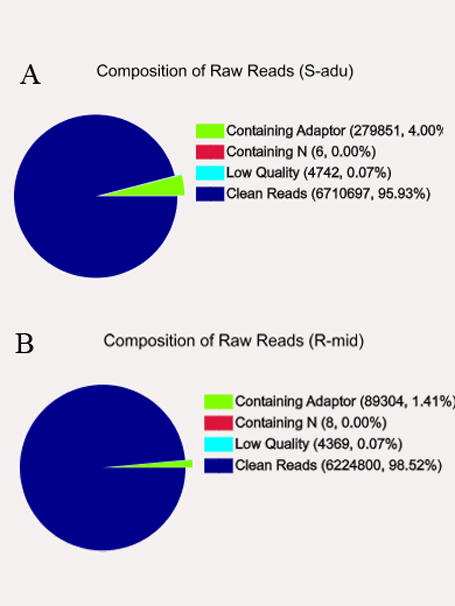

Supplement: Figure S2 — The quality of Propylaea japonica DGE sequences. (A) SUS, (B) R-mid. (TIF) [file pone.0100946.s002.tif]

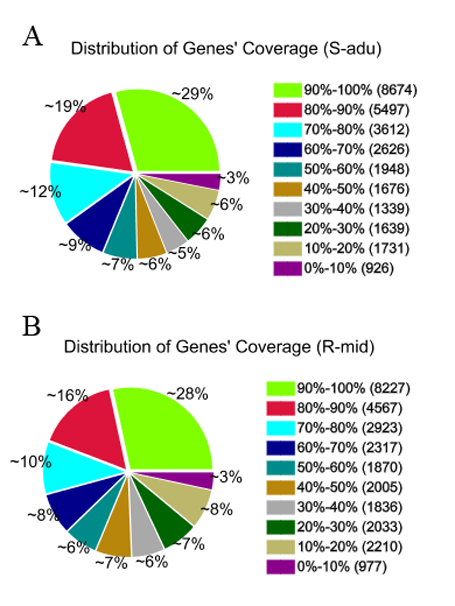

Supplement: Figure S3 — The distribution of gene coverage of Propylaea japonica DGE sequences. (A) SUS, (B) R-mid. (TIF) [file pone.0100946.s003.tif]
