# Supplementary material for: De Novo Sequencing-Based Transcriptome and Digital Gene Expression Analysis Reveals Insecticide Resistance-Relevant Genes in Propylaea japonica (Thunberg) (Coleoptea: Coccinellidae)
Source: PLoS One. 2014 Jun 24;9(6):e100946. doi: 10.1371/journal.pone.0100946 (PMC4069172; doi:10.1371/journal.pone.0100946)
Supplement: Table S1 — The primer of qPCR. (DOC) [file pone.0100946.s007.doc]

Table S1 The primer of qPCR.

| Primer name | Sequence (5'to3') |
| --- | --- |
| CL500- housekeep- QP -F | 5'-GTTACTCTTTCACCACCACA-3' |
| CL500- housekeep- QP -R | 5'-GGGCAACGGAATCTTT-3' |
| CL3277-U- QP -F | 5'-TGCCATACAGACGACTTG-3' |
| CL3277-U- QP -R | 5'-TCCTTCATTGTCCTCCA-3' |
| CL2039-U- QP -F | 5'-ATGTGGGAAATCAAAGC-3' |
| CL2039-U- QP -R | 5'-CACCCGGATGGGACTA-3' |
| Unigene1069-U-QP-F | 5'-TTATTTCCCAAACAGACC-3' |
| Unigene1069-U- QP -R | 5'-GGAGCGATTTCCATTC-3' |
| Unigene19166-U- QP -F | 5'-CCCAGACAGGTTTAGAGG-3' |
| Unigene19166-U- QP -R | 5'-TTTCGGTCTTGGCACA-3' |
| Unigene556-U- QP -F | 5'-GACATCCGTTTGCCTAT-3' |
| Unigene556-U- QP -R | 5'-TTGATAATGGTGGACAGC-3' |
| Unigene1568-U- QP -F | 5'-ATTTGCGAAGAGGTTGA-3' |
| Unigene1568-U- QP -R | 5'-CGTAAGATGGGTGTTCAT-3' |
| Unigene15877-U- QP -F | 5'-TTCTTAGCGGGTTTCG-3' |
| Unigene15877-U- QP -R | 5'-ATGGCTTCATAAGTAATCCTAC-3' |
| Unigene21907-U- QP -F | 5'-AGAGCCCAACTATGAAGC-3' |
| Unigene21907-U- QP -R | 5'-GATATGCCCACGGAGC-3' |
| Unigene21058-U- QP -F | 5'-ACCTCAAAGACCCTTATTACC-3' |
| Unigene21058-U- QP -R | 5'-TCTCCACAGCCCGAAA-3' |
| Unigene12340-U- QP -F | 5'-TTTGACTGAACCCAGCGTAG-3' |
| Unigene12340-U- QP -R | 5'-GGCGAAACCAATCGTATGT-3' |
| Unigene160-U- QP -F | 5'-GATGCCAGTTTCCCTAT-3' |
| Unigene160-U- QP -R | 5'-TTTCTATCTTTCCCTCCA-3' |
| Unigene21647-U- QP -F | 5'-CAGGTGGAGCGTCAGTT-3' |
| Unigene21647-U- QP -R | 5'-TTGGGCTATTGCTTTGTG-3' |
| Unigene10291-U- QP -F | 5'-GATGGGACTTTGGTGGAA-3' |
| Unigene10291-U- QP -R | 5'-TTGTGGAGGATACAGGTGAG-3' |
| Unigene9996-D- QP -F | 5'-ATTTCGCTTCTTTGCC-3' |
| Unigene9996-D- QP -R | 5'-CAGAGGTTCGTGGTGAT-3' |
| Unigene17900-D- QP -F | 5'-AAAATGGCCGCTAAGTTGATC-3' |
| Unigene17900-D- QP -R | 5'-TTGTATGGCTTGGGTGGGT-3' |
| Unigene9705-D- QP -F | 5'-GTCAATTTGATAGTACCTTGTG-3' |
| Unigene9705-D- QP -R | 5'-TGATGGGATGATTTCTG-3' |
