# Supplementary material for: De Novo Sequencing-Based Transcriptome and Digital Gene Expression Analysis Reveals Insecticide Resistance-Relevant Genes in Propylaea japonica (Thunberg) (Coleoptea: Coccinellidae)
Source: PLoS One. 2014 Jun 24;9(6):e100946. doi: 10.1371/journal.pone.0100946 (PMC4069172; doi:10.1371/journal.pone.0100946)
Supplement: Table S2 — The unigene metabolic pathway analysis of Propylaea japonica was conducted using the Kyoto Encyclopedia of Genes and Genomes (KEGG) annotation system. (DOC) [file pone.0100946.s008.doc]

Table S2 The unigene metabolic pathway analysis of *Propylaea japonica* was conducted using the Kyoto Encyclopedia of Genes and Genomes (KEGG) annotation system

| **Code** | **Pathway** | **All genes with pathway annotation (16147)** | **Pathway ID** |
| --- | --- | --- | --- |
| 1 | Metabolic pathways | 2276 (14.1%) | ko01100 |
| 2 | RNA transport | 548 (3.39%) | ko03013 |
| 3 | Purine metabolism | 540 (3.34%) | ko00230 |
| 4 | Regulation of actin cytoskeleton | 530 (3.28%) | ko04810 |
| 5 | Focal adhesion | 516 (3.2%) | ko04510 |
| 6 | Pathways in cancer | 512 (3.17%) | ko05200 |
| 7 | Huntington's disease | 482 (2.99%) | ko05016 |
| 8 | Spliceosome | 468 (2.9%) | ko03040 |
| 9 | Epstein-Barr virus infection | 433 (2.68%) | ko05169 |
| 10 | Bile secretion | 427 (2.64%) | ko04976 |
| 11 | Ubiquitin mediated proteolysis | 399 (2.47%) | ko04120 |
| 12 | Vascular smooth muscle contraction | 391 (2.42%) | ko04270 |
| 13 | Endocytosis | 382 (2.37%) | ko04144 |
| 14 | HTLV-I infection | 379 (2.35%) | ko05166 |
| 15 | Tight junction | 372 (2.3%) | ko04530 |
| 16 | Pyrimidine metabolism | 356 (2.2%) | ko00240 |
| 17 | Protein processing in endoplasmic reticulum | 347 (2.15%) | ko04141 |
| 18 | MAPK signaling pathway | 341 (2.11%) | ko04010 |
| 19 | Phagosome | 324 (2.01%) | ko04145 |
| 20 | Lysosome | 324 (2.01%) | ko04142 |
| 21 | Insulin signaling pathway | 317 (1.96%) | ko04910 |
| 22 | Transcriptional misregulation in cancer | 313 (1.94%) | ko05202 |
| 23 | Amoebiasis | 304 (1.88%) | ko05146 |
| 24 | Influenza A | 301 (1.86%) | ko05164 |
| 25 | Calcium signaling pathway | 301 (1.86%) | ko04020 |
| 26 | mRNA surveillance pathway | 301 (1.86%) | ko03015 |
| 27 | Cell cycle | 297 (1.84%) | ko04110 |
| 28 | Dilated cardiomyopathy | 292 (1.81%) | ko05414 |
| 29 | ABC transporters | 291 (1.8%) | ko02010 |
| 30 | Alzheimer's disease | 290 (1.8%) | ko05010 |
| 31 | Ribosome | 279 (1.73%) | ko03010 |
| 32 | Pancreatic secretion | 278 (1.72%) | ko04972 |
| 33 | Salmonella infection | 278 (1.72%) | ko05132 |
| 34 | Neuroactive ligand-receptor interaction | 277 (1.72%) | ko04080 |
| 35 | Hypertrophic cardiomyopathy (HCM) | 273 (1.69%) | ko05410 |
| 36 | Axon guidance | 269 (1.67%) | ko04360 |
| 37 | Ribosome biogenesis in eukaryotes | 267 (1.65%) | ko03008 |
| 38 | Herpes simplex infection | 266 (1.65%) | ko05168 |
| 39 | Chemokine signaling pathway | 263 (1.63%) | ko04062 |
| 40 | Wnt signaling pathway | 254 (1.57%) | ko04310 |
| 41 | Gastric acid secretion | 234 (1.45%) | ko04971 |
| 42 | Tuberculosis | 227 (1.41%) | ko05152 |
| 43 | Vibrio cholerae infection | 226 (1.4%) | ko05110 |
| 44 | Protein digestion and absorption | 225 (1.39%) | ko04974 |
| 45 | ECM-receptor interaction | 222 (1.37%) | ko04512 |
| 46 | Adherens junction | 222 (1.37%) | ko04520 |
| 47 | Pathogenic Escherichia coli infection | 221 (1.37%) | ko05130 |
| 48 | Progesterone-mediated oocyte maturation | 212 (1.31%) | ko04914 |
| 49 | RNA polymerase | 212 (1.31%) | ko03020 |
| 50 | Starch and sucrose metabolism | 210 (1.3%) | ko00500 |
| 51 | Fc gamma R-mediated phagocytosis | 208 (1.29%) | ko04666 |
| 52 | Salivary secretion | 208 (1.29%) | ko04970 |
| 53 | Peroxisome | 205 (1.27%) | ko04146 |
| 54 | Oocyte meiosis | 198 (1.23%) | ko04114 |
| 55 | Bacterial invasion of epithelial cells | 196 (1.21%) | ko05100 |
| 56 | Leukocyte transendothelial migration | 195 (1.21%) | ko04670 |
| 57 | Toxoplasmosis | 192 (1.19%) | ko05145 |
| 58 | Cardiac muscle contraction | 191 (1.18%) | ko04260 |
| 59 | Viral myocarditis | 191 (1.18%) | ko05416 |
| 60 | Glycerophospholipid metabolism | 190 (1.18%) | ko00564 |
| 61 | Drug metabolism - other enzymes | 186 (1.15%) | ko00983 |
| 62 | Neurotrophin signaling pathway | 182 (1.13%) | ko04722 |
| 63 | Lysine degradation | 180 (1.11%) | ko00310 |
| 64 | RNA degradation | 180 (1.11%) | ko03018 |
| 65 | Phosphatidylinositol signaling system | 177 (1.1%) | ko04070 |
| 66 | Vasopressin-regulated water reabsorption | 174 (1.08%) | ko04962 |
| 67 | Glutamatergic synapse | 173 (1.07%) | ko04724 |
| 68 | T cell receptor signaling pathway | 168 (1.04%) | ko04660 |
| 69 | Mineral absorption | 166 (1.03%) | ko04978 |
| 70 | Oxidative phosphorylation | 166 (1.03%) | ko00190 |
| 71 | Parkinson's disease | 164 (1.02%) | ko05012 |
| 72 | GnRH signaling pathway | 162 (1%) | ko04912 |
| 73 | Dopaminergic synapse | 160 (0.99%) | ko04728 |
| 74 | Amino sugar and nucleotide sugar metabolism | 156 (0.97%) | ko00520 |
| 75 | Small cell lung cancer | 155 (0.96%) | ko05222 |
| 76 | Melanogenesis | 151 (0.94%) | ko04916 |
| 77 | Shigellosis | 151 (0.94%) | ko05131 |
| 78 | Measles | 150 (0.93%) | ko05162 |
| 79 | Glycerolipid metabolism | 149 (0.92%) | ko00561 |
| 80 | Pentose and glucuronate interconversions | 148 (0.92%) | ko00040 |
| 81 | Amyotrophic lateral sclerosis (ALS) | 148 (0.92%) | ko05014 |
| 82 | Alcoholism | 146 (0.9%) | ko05034 |
| 83 | Gap junction | 145 (0.9%) | ko04540 |
| 84 | Dorso-ventral axis formation | 144 (0.89%) | ko04320 |
| 85 | Glycine, serine and threonine metabolism | 143 (0.89%) | ko00260 |
| 86 | Cell adhesion molecules (CAMs) | 143 (0.89%) | ko04514 |
| 87 | Prostate cancer | 142 (0.88%) | ko05215 |
| 88 | ErbB signaling pathway | 140 (0.87%) | ko04012 |
| 89 | Glycolysis / Gluconeogenesis | 135 (0.84%) | ko00010 |
| 90 | Hepatitis C | 131 (0.81%) | ko05160 |
| 91 | Inositol phosphate metabolism | 131 (0.81%) | ko00562 |
| 92 | Aminoacyl-tRNA biosynthesis | 129 (0.8%) | ko00970 |
| 93 | Renal cell carcinoma | 128 (0.79%) | ko05211 |
| 94 | Metabolism of xenobiotics by cytochrome P450 | 128 (0.79%) | ko00980 |
| 95 | Cholinergic synapse | 125 (0.77%) | ko04725 |
| 96 | Morphine addiction | 125 (0.77%) | ko05032 |
| 97 | Carbohydrate digestion and absorption | 125 (0.77%) | ko04973 |
| 98 | Retrograde endocannabinoid signaling | 124 (0.77%) | ko04723 |
| 99 | TGF-beta signaling pathway | 124 (0.77%) | ko04350 |
| 100 | Retinol metabolism | 123 (0.76%) | ko00830 |
| 101 | VEGF signaling pathway | 123 (0.76%) | ko04370 |
| 102 | PPAR signaling pathway | 122 (0.76%) | ko03320 |
| 103 | Drug metabolism - cytochrome P450 | 121 (0.75%) | ko00982 |
| 104 | GABAergic synapse | 121 (0.75%) | ko04727 |
| 105 | Cytosolic DNA-sensing pathway | 120 (0.74%) | ko04623 |
| 106 | Basal transcription factors | 120 (0.74%) | ko03022 |
| 107 | Synaptic vesicle cycle | 120 (0.74%) | ko04721 |
| 108 | Porphyrin and chlorophyll metabolism | 119 (0.74%) | ko00860 |
| 109 | Chagas disease (American trypanosomiasis) | 119 (0.74%) | ko05142 |
| 110 | Endocrine and other factor-regulated calcium reabsorption | 119 (0.74%) | ko04961 |
| 111 | Epithelial cell signaling in Helicobacter pylori infection | 119 (0.74%) | ko05120 |
| 112 | Long-term potentiation | 118 (0.73%) | ko04720 |
| 113 | Glutathione metabolism | 115 (0.71%) | ko00480 |
| 114 | Galactose metabolism | 115 (0.71%) | ko00052 |
| 115 | Other types of O-glycan biosynthesis | 115 (0.71%) | ko00514 |
| 116 | Fanconi anemia pathway | 114 (0.71%) | ko03460 |
| 117 | Nucleotide excision repair | 113 (0.7%) | ko03420 |
| 118 | Legionellosis | 113 (0.7%) | ko05134 |
| 119 | Adipocytokine signaling pathway | 113 (0.7%) | ko04920 |
| 120 | Arrhythmogenic right ventricular cardiomyopathy (ARVC) | 113 (0.7%) | ko05412 |
| 121 | Pyruvate metabolism | 112 (0.69%) | ko00620 |
| 122 | Vitamin digestion and absorption | 110 (0.68%) | ko04977 |
| 123 | Arginine and proline metabolism | 109 (0.68%) | ko00330 |
| 124 | Toll-like receptor signaling pathway | 107 (0.66%) | ko04620 |
| 125 | Tyrosine metabolism | 107 (0.66%) | ko00350 |
| 126 | Serotonergic synapse | 107 (0.66%) | ko04726 |
| 127 | Steroid hormone biosynthesis | 107 (0.66%) | ko00140 |
| 128 | mTOR signaling pathway | 106 (0.66%) | ko04150 |
| 129 | Fat digestion and absorption | 106 (0.66%) | ko04975 |
| 130 | Glioma | 106 (0.66%) | ko05214 |
| 131 | Osteoclast differentiation | 105 (0.65%) | ko04380 |
| 132 | Hedgehog signaling pathway | 105 (0.65%) | ko04340 |
| 133 | Ascorbate and aldarate metabolism | 105 (0.65%) | ko00053 |
| 134 | Rheumatoid arthritis | 104 (0.64%) | ko05323 |
| 135 | Notch signaling pathway | 103 (0.64%) | ko04330 |
| 136 | p53 signaling pathway | 102 (0.63%) | ko04115 |
| 137 | Colorectal cancer | 102 (0.63%) | ko05210 |
| 138 | Complement and coagulation cascades | 102 (0.63%) | ko04610 |
| 139 | DNA replication | 101 (0.63%) | ko03030 |
| 140 | Chronic myeloid leukemia | 101 (0.63%) | ko05220 |
| 141 | Cysteine and methionine metabolism | 100 (0.62%) | ko00270 |
| 142 | Type II diabetes mellitus | 98 (0.61%) | ko04930 |
| 143 | Antigen processing and presentation | 98 (0.61%) | ko04612 |
| 144 | Prion diseases | 97 (0.6%) | ko05020 |
| 145 | Jak-STAT signaling pathway | 96 (0.59%) | ko04630 |
| 146 | Phototransduction - fly | 96 (0.59%) | ko04745 |
| 147 | Fc epsilon RI signaling pathway | 95 (0.59%) | ko04664 |
| 148 | Endometrial cancer | 95 (0.59%) | ko05213 |
| 149 | Amphetamine addiction | 92 (0.57%) | ko05031 |
| 150 | B cell receptor signaling pathway | 91 (0.56%) | ko04662 |
| 151 | Natural killer cell mediated cytotoxicity | 89 (0.55%) | ko04650 |
| 152 | Pentose phosphate pathway | 89 (0.55%) | ko00030 |
| 153 | NF-kappa B signaling pathway | 89 (0.55%) | ko04064 |
| 154 | Fatty acid metabolism | 88 (0.54%) | ko00071 |
| 155 | Basal cell carcinoma | 87 (0.54%) | ko05217 |
| 156 | Valine, leucine and isoleucine degradation | 84 (0.52%) | ko00280 |
| 157 | Pancreatic cancer | 84 (0.52%) | ko05212 |
| 158 | Hematopoietic cell lineage | 82 (0.51%) | ko04640 |
| 159 | Long-term depression | 81 (0.5%) | ko04730 |
| 160 | Tryptophan metabolism | 80 (0.5%) | ko00380 |
| 161 | Cytokine-cytokine receptor interaction | 80 (0.5%) | ko04060 |
| 162 | Insect hormone biosynthesis | 79 (0.49%) | ko00981 |
| 163 | Apoptosis | 78 (0.48%) | ko04210 |
| 164 | Fructose and mannose metabolism | 78 (0.48%) | ko00051 |
| 165 | Cocaine addiction | 76 (0.47%) | ko05030 |
| 166 | Alanine, aspartate and glutamate metabolism | 76 (0.47%) | ko00250 |
| 167 | N-Glycan biosynthesis | 74 (0.46%) | ko00510 |
| 168 | Acute myeloid leukemia | 74 (0.46%) | ko05221 |
| 169 | Citrate cycle (TCA cycle) | 74 (0.46%) | ko00020 |
| 170 | Olfactory transduction | 74 (0.46%) | ko04740 |
| 171 | Pertussis | 72 (0.45%) | ko05133 |
| 172 | Aldosterone-regulated sodium reabsorption | 72 (0.45%) | ko04960 |
| 173 | alpha-Linolenic acid metabolism | 72 (0.45%) | ko00592 |
| 174 | Base excision repair | 71 (0.44%) | ko03410 |
| 175 | Glycosaminoglycan biosynthesis - heparan sulfate | 69 (0.43%) | ko00534 |
| 176 | Malaria | 68 (0.42%) | ko05144 |
| 177 | beta-Alanine metabolism | 68 (0.42%) | ko00410 |
| 178 | Propanoate metabolism | 66 (0.41%) | ko00640 |
| 179 | Non-small cell lung cancer | 62 (0.38%) | ko05223 |
| 180 | Homologous recombination | 62 (0.38%) | ko03440 |
| 181 | Staphylococcus aureus infection | 61 (0.38%) | ko05150 |
| 182 | Melanoma | 60 (0.37%) | ko05218 |
| 183 | Butanoate metabolism | 60 (0.37%) | ko00650 |
| 184 | Mismatch repair | 58 (0.36%) | ko03430 |
| 185 | Proteasome | 58 (0.36%) | ko03050 |
| 186 | Leishmaniasis | 57 (0.35%) | ko05140 |
| 187 | MAPK signaling pathway - fly | 57 (0.35%) | ko04013 |
| 188 | Glycosylphosphatidylinositol(GPI)-anchor biosynthesis | 55 (0.34%) | ko00563 |
| 189 | Phenylalanine metabolism | 55 (0.34%) | ko00360 |
| 190 | Phototransduction | 54 (0.33%) | ko04744 |
| 191 | Terpenoid backbone biosynthesis | 54 (0.33%) | ko00900 |
| 192 | Proximal tubule bicarbonate reclamation | 54 (0.33%) | ko04964 |
| 193 | Renin-angiotensin system | 54 (0.33%) | ko04614 |
| 194 | NOD-like receptor signaling pathway | 53 (0.33%) | ko04621 |
| 195 | Sphingolipid metabolism | 53 (0.33%) | ko00600 |
| 196 | Arachidonic acid metabolism | 50 (0.31%) | ko00590 |
| 197 | Nicotinate and nicotinamide metabolism | 50 (0.31%) | ko00760 |
| 198 | Ether lipid metabolism | 49 (0.3%) | ko00565 |
| 199 | Biosynthesis of unsaturated fatty acids | 48 (0.3%) | ko01040 |
| 200 | Collecting duct acid secretion | 48 (0.3%) | ko04966 |
| 201 | Thyroid cancer | 48 (0.3%) | ko05216 |
| 202 | Fatty acid biosynthesis | 46 (0.28%) | ko00061 |
| 203 | Glyoxylate and dicarboxylate metabolism | 46 (0.28%) | ko00630 |
| 204 | Primary immunodeficiency | 45 (0.28%) | ko05340 |
| 205 | Histidine metabolism | 43 (0.27%) | ko00340 |
| 206 | Nicotine addiction | 42 (0.26%) | ko05033 |
| 207 | RIG-I-like receptor signaling pathway | 42 (0.26%) | ko04622 |
| 208 | Fatty acid elongation | 41 (0.25%) | ko00062 |
| 209 | Riboflavin metabolism | 40 (0.25%) | ko00740 |
| 210 | Systemic lupus erythematosus | 39 (0.24%) | ko05322 |
| 211 | Bladder cancer | 39 (0.24%) | ko05219 |
| 212 | Protein export | 38 (0.24%) | ko03060 |
| 213 | Steroid biosynthesis | 38 (0.24%) | ko00100 |
| 214 | Non-homologous end-joining | 37 (0.23%) | ko03450 |
| 215 | Glycosaminoglycan degradation | 35 (0.22%) | ko00531 |
| 216 | Selenocompound metabolism | 34 (0.21%) | ko00450 |
| 217 | Circadian rhythm - fly | 34 (0.21%) | ko04711 |
| 218 | Other glycan degradation | 33 (0.2%) | ko00511 |
| 219 | Taste transduction | 33 (0.2%) | ko04742 |
| 220 | Linoleic acid metabolism | 32 (0.2%) | ko00591 |
| 221 | Regulation of autophagy | 30 (0.19%) | ko04140 |
| 222 | Mucin type O-Glycan biosynthesis | 30 (0.19%) | ko00512 |
| 223 | Pantothenate and CoA biosynthesis | 30 (0.19%) | ko00770 |
| 224 | SNARE interactions in vesicular transport | 30 (0.19%) | ko04130 |
| 225 | Ubiquinone and other terpenoid-quinone biosynthesis | 27 (0.17%) | ko00130 |
| 226 | Circadian rhythm - mammal | 26 (0.16%) | ko04710 |
| 227 | One carbon pool by folate | 26 (0.16%) | ko00670 |
| 228 | Sulfur metabolism | 24 (0.15%) | ko00920 |
| 229 | Caffeine metabolism | 24 (0.15%) | ko00232 |
| 230 | Folate biosynthesis | 24 (0.15%) | ko00790 |
| 231 | Autoimmune thyroid disease | 23 (0.14%) | ko05320 |
| 232 | African trypanosomiasis | 23 (0.14%) | ko05143 |
| 233 | Cyanoamino acid metabolism | 23 (0.14%) | ko00460 |
| 234 | Taurine and hypotaurine metabolism | 21 (0.13%) | ko00430 |
| 235 | Glycosaminoglycan biosynthesis - chondroitin sulfate | 19 (0.12%) | ko00532 |
| 236 | Type I diabetes mellitus | 18 (0.11%) | ko04940 |
| 237 | Sulfur relay system | 16 (0.1%) | ko04122 |
| 238 | Glycosphingolipid biosynthesis - globo series | 16 (0.1%) | ko00603 |
| 239 | Maturity onset diabetes of the young | 16 (0.1%) | ko04950 |
| 240 | Valine, leucine and isoleucine biosynthesis | 16 (0.1%) | ko00290 |
| 241 | D-Arginine and D-ornithine metabolism | 12 (0.07%) | ko00472 |
| 242 | Synthesis and degradation of ketone bodies | 12 (0.07%) | ko00072 |
| 243 | Glycosaminoglycan biosynthesis - keratan sulfate | 11 (0.07%) | ko00533 |
| 244 | Glycosphingolipid biosynthesis - ganglio series | 11 (0.07%) | ko00604 |
| 245 | Primary bile acid biosynthesis | 11 (0.07%) | ko00120 |
| 246 | Butirosin and neomycin biosynthesis | 11 (0.07%) | ko00524 |
| 247 | Glycosphingolipid biosynthesis - lacto and neolacto series | 10 (0.06%) | ko00601 |
| 248 | Lipoic acid metabolism | 8 (0.05%) | ko00785 |
| 249 | Thiamine metabolism | 7 (0.04%) | ko00730 |
| 250 | Biotin metabolism | 7 (0.04%) | ko00780 |
| 251 | D-Glutamine and D-glutamate metabolism | 5 (0.03%) | ko00471 |
| 252 | Phenylalanine, tyrosine and tryptophan biosynthesis | 5 (0.03%) | ko00400 |
| 253 | Vitamin B6 metabolism | 5 (0.03%) | ko00750 |
| 254 | Lysine biosynthesis | 4 (0.02%) | ko00300 |
| 255 | Asthma | 4 (0.02%) | ko05310 |
