# Supplementary material for: De Novo Sequencing-Based Transcriptome and Digital Gene Expression Analysis Reveals Insecticide Resistance-Relevant Genes in Propylaea japonica (Thunberg) (Coleoptea: Coccinellidae)
Source: PLoS One. 2014 Jun 24;9(6):e100946. doi: 10.1371/journal.pone.0100946 (PMC4069172; doi:10.1371/journal.pone.0100946)
Supplement: Table S3 — Predicted single nucleotide polymorphisms (SNP) in Propylaea japonica sequences. (DOC) [file pone.0100946.s009.doc]

Table S3 Predicted single nucleotide polymorphisms (SNP) in *Propylaea japonica* sequences.

| SNP Type | Counts |
| --- | --- |
| Transition | 85,056 |
| A-G | 43,259 |
| C-T | 41,797 |
| Transversion | 43,837 |
| A-C | 9,820 |
| A-T | 17,220 |
| C-G | 6,636 |
| G-T | 10,161 |
| Total | 128,893 |
