# Supplementary material for: De Novo Sequencing-Based Transcriptome and Digital Gene Expression Analysis Reveals Insecticide Resistance-Relevant Genes in Propylaea japonica (Thunberg) (Coleoptea: Coccinellidae)
Source: PLoS One. 2014 Jun 24;9(6):e100946. doi: 10.1371/journal.pone.0100946 (PMC4069172; doi:10.1371/journal.pone.0100946)
Supplement: Table S4 — Summary of microsatellite loci predicted in Propylaea japonica sequences. (DOC) [file pone.0100946.s010.doc]

Table S4 Summary of microsatellite loci predicted in *Propylaea japonica* sequences.

| Numer of repeats | Mono-  nucleotde repeats | Di-  nucleoide repeats | Tri-  nucletide repeats | Quad-  nucletide repeat | Penta-  nucletide repeats | Hexa-  nuclotide repeats |
| --- | --- | --- | --- | --- | --- | --- |
| 4 | 0 | 0 | 0 | 0 | 3 | 5 |
| 5 | 0 | 0 | 220 | 3 | 2 | 0 |
| 6 | 0 | 18 | 26 | 2 | 0 | 0 |
| 7 | 0 | 17 | 5 | 1 | 0 | 0 |
| 8 | 0 | 2 | 1 | 0 | 0 | 0 |
| 9 | 0 | 5 | 0 | 0 | 0 | 0 |
| 10 | 0 | 1 | 0 | 0 | 0 | 0 |
| 11 | 0 | 2 | 0 | 0 | 0 | 0 |
| 12 | 10 | 1 | 1 | 0 | 0 | 0 |
| 13 | 5 | 0 | 0 | 0 | 0 | 0 |
| 14 | 6 | 0 | 0 | 0 | 0 | 0 |
| 15 | 3 | 0 | 0 | 0 | 0 | 0 |
| 16 | 1 | 0 | 0 | 0 | 0 | 0 |
| 18 | 1 | 0 | 0 | 0 | 0 | 0 |
| 19 | 1 | 0 | 0 | 0 | 0 | 0 |
| 21 | 3 | 0 | 0 | 0 | 0 | 0 |
| 22 | 4 | 0 | 0 | 0 | 0 | 0 |
| 23 | 5 | 0 | 0 | 0 | 0 | 0 |
| 24 | 1 | 0 | 0 | 0 | 0 | 0 |
| 25 | 0 | 1 | 0 | 0 | 0 | 0 |
| Sub  Total | 40 | 47 | 253 | 6 | 5 | 5 |
