# Supplementary material for: De Novo Sequencing-Based Transcriptome and Digital Gene Expression Analysis Reveals Insecticide Resistance-Relevant Genes in Propylaea japonica (Thunberg) (Coleoptea: Coccinellidae)
Source: PLoS One. 2014 Jun 24;9(6):e100946. doi: 10.1371/journal.pone.0100946 (PMC4069172; doi:10.1371/journal.pone.0100946)
Supplement: Table S6 — Representatives of putative insecticide resistant genes as predicted by DGE. Limitations of all significantly different expressed genes between R-low (or R-mid) and SUS are based on FDR≤0.001 and the absolute value of log2Ratio≥1. The log2Ratio(R-mid/SUS) indicates the change of gene expression; a positive number means up-regulation and a negative one means down-regulation. (DOC) [file pone.0100946.s012.doc]

Table S6 Representatives of putative insecticide resistant genes as predicted by DGE. Limitations of all signiﬁcantly different expressed genes between R-low (or R-mid) and SUS are based on FDR≤0.001 and the absolute value of log2Ratio≥1. The log2Ratio(R-mid/SUS) indicates the change of gene expression; a positive number means up-regulation and a negative one means down-regulation.

| Gene ID  (Unigene-) | Length | RPKM  -SUS | RPKM  -R-mid | Log2Ratio (R-mid/SUS) | Annotation |
| --- | --- | --- | --- | --- | --- |
| 9043 | 285 | 0.01 | 5.80 | 9.18 | cytochrome P450 345D1 |
| 6019 | 285 | 0.01 | 1.66 | 7.37 | cytochrome P450 307A1 |
| 16545 | 265 | 0.01 | 0.89 | 6.48 | Cytochrome P450 4c3 |
| 18466 | 298 | 0.01 | 0.79 | 6.31 | cytochrome P450 CYP349b1 |
| 5087 | 268 | 4.80 | 19.39 | 2.01 | similar to cytochrome P450 CYP6BK17 |
| 25667 | 246 | 2.62 | 7.68 | 1.55 | cytochrome P450, putative |
| 18465 | 476 | 2.25 | 5.96 | 1.40 | cytochrome P450 CYP349b1 |
| CL999.  Contig3 | 543 | 0.79 | 1.74 | 1.14 | cytochrome P450 349A1 |
| 12019 | 439 | 2.93 | 33.90 | 3.63 | cytochrome P450-like protein |
| 18631 | 686 | 0.63 | 2.75 | 2.14 | cytochrome P450 307A1 |
| 22607 | 295 | 0.01 | 0.80 | 6.32 | cytochrome P450-like protein |
| CL2039.  Contig1 | 1661 | 0.01 | 0.57 | 5.83 | cytochrome P450-like protein |
| 26285 | 287 | 1.50 | 8.23 | 2.46 | cytochrome P450 CYP4AB2 |
| 18946 | 519 | 4.55 | 22.30 | 2.29 | cytochrome P450, family 4, subfamily Q, polypeptide 7 |
| 9578 | 310 | 0.69 | 8.38 | 3.60 | cytochrome P450 monooxigenase CYP4Q1 |
| 16033 | 374 | 0.57 | 0.63 | 0.14 | cytochrome P450 6BS1 |
| 1568 | 1774 | 8.47 | 41.67 | 2.30 | cytochrome P450 monooxigenase CYP4Q2 |
| CL3586.  Contig1 | 1225 | 15.24 | 91.40 | 2.58 | cytochrome P450 9Z6 |
| 15786 | 402 | 0.53 | 1.76 | 1.72 | cytochrome P450 CYP6BS2 |
| 7454 | 270 | 0.79 | 4.37 | 2.46 | antennae-rich cytochrome P450 |
| 4279 | 299 | 2.87 | 1.58 | -0.86 | cytochrome P450-like protein |
| 8806 | 204 | 6.31 | 0.01 | -9.30 | cytochrome P450 4C1-like |
| 17927 | 255 | 13.46 | 29.64 | 1.14 | cytochrome P450 305A1 |
| 13436 | 293 | 4.39 | 1.61 | -1.45 | cytochrome P450-like protein |
| 9860 | 252 | 5.11 | 16.87 | 1.72 | Probable cytochrome P450 6g2 |
| 13683 | 484 | 14.19 | 22.45 | 0.66 | cytochrome P450 345A1 |
| CL2039  Contig3 | 1678 | 1.41 | 1.27 | -0.15 | cytochrome P450-like protein |
| 12341 | 226 | 3.80 | 17.77 | 2.23 | microsomal glutathione S-transferase 1 |
| 18040 | 332 | 6.46 | 12.10 | 0.90 | glutathione S-transferase C-terminal domain-containing protein |
| 12340 | 264 | 11.38 | 23.26 | 1.03 | similar to microsomal glutathione s-transferase |
| 7981 | 249 | 0.86 | 0.95 | 0.14 | nicotinic acetylcholine receptor alpha 2 |
| CL3277.  Contig1 | 1091 | 0.01 | 5.63 | 9.14 | cytochrome P450 345B1 |
| 1069 | 762 | 9.29 | 155.00 | 4.06 | similar to cytochrome P450 |
| CL999.  Contig4 | 430 | 2.49 | 33.51 | 3.75 | similar to cytochrome P450 |
| 19166 | 979 | 22.37 | 246.11 | 3.46 | cytochrome P450 |
| CL3586.  Contig1 | 1225 | 15.24 | 91.40 | 2.58 | cytochrome P450 9Z6 |
| 556 | 1317 | 7.66 | 45.02 | 2.56 | cytochrome P450 monooxigenase CYP4Q2 |
| 1568 | 1774 | 8.47 | 41.68 | 2.30 | cytochrome P450 monooxigenase CYP4Q2 |
| 18946 | 519 | 4.55 | 22.30 | 2.29 | similar to cytochrome P450 |
| 5340 | 370 | 4.64 | 14.68 | 1.66 | cytochrome P450 monooxygenase Cyp4M5 |
| 15877 | 530 | 2.02 | 15.15 | 2.90 | cytochrome P450 6BQ13 |
| CL1929.  Contig1 | 620 | 6.58 | 44.58 | 2.76 | Chymotrypsin/elastase isoinhibitors 2 to 5 |
| 21908 | 410 | 7.33 | 37.45 | 2.36 | cytochrome P450-like protein |
| 21907 | 524 | 6.14 | 31.11 | 2.34 | cytochrome P450 isoform 9F2 |
| 21564 | 1334 | 11.58 | 47.63 | 2.04 | cytochrome P450 CYP18A1 |
| 22514 | 312 | 14.44 | 58.29 | 2.01 | cytochrome P450-like protein |
| 11865 | 312 | 35.76 | 131.74 | 1.88 | cytochrome P450 9Z4 |
| 23897 | 1568 | 76.64 | 267.41 | 1.80 | cytochrome P450 345D2 |
| 8214 | 368 | 20.99 | 73.18 | 1.80 | similar to Cytochrome P450 315a1, mitochondrial precursor |
| 11833 | 399 | 60.23 | 208.39 | 1.79 | cytochrome P450 CYP9z23 |
| CL334.  Contig1 | 449 | 28.19 | 90.49 | 1.68 | juvenile hormone esterase isoform B |
| 14663 | 282 | 67.72 | 211.93 | 1.65 | cytochrome P450 isoform 9F2 |
| 22515 | 986 | 23.72 | 70.91 | 1.58 | cytochrome P450-like protein |
| 21058 | 930 | 22.61 | 64.52 | 1.51 | similar to glutathione S-transferase |
| 21074 | 1328 | 244.31 | 691.24 | 1.50 | cytochrome P450 9Z4 |
| 10530 | 655 | 110.40 | 297.53 | 1.43 | cytochrome P450 CYP9Z1 |
| 9172 | 602 | 23.88 | 56.11 | 1.23 | similar to Cytochrome P450 315a1, mitochondrial precursor (CYPCCCXVA1) (Protein shadow) |
| 7797 | 233 | 2.76 | 2.03 | -0.45 | similar to cytochrome P450 |
| CL999.  Contig1 | 202 | 2.12 | 3.51 | 0.72 | cytochrome P450 CYP349b1 |
| 1642 | 801 | 0.80 | 0.01 | -6.33 | cytochrome P450 303A1 |
| CL2039  Contig2 | 1740 | 0.12 | 0.01 | -3.62 | cytochrome P450-like protein |
| 6935 | 229 | 8.43 | 11.35 | 0.42 | similar to Cytochrome P450 315a1, mitochondrial precursor |
| 21893 | 653 | 1.64 | 0.36 | -2.18 | cytochrome P450 6BQ4 |
| 9996 | 249 | 4.31 | 0.01 | -8.75 | cytochrome P450 301B1 |
| 5484 | 330 | 26.66 | 26.48 | -0.01 | cytochrome P450 monooxygenase |
| CL1447.  Contig1 | 201 | 11.74 | 11.75 | 0.001 | cytochrome P450 CYP314A1 |
| 7021 | 269 | 2.39 | 0.01 | -7.90 | cytochrome P450 49A1 |
| 13643 | 297 | 6.50 | 10.34 | 0.67 | cytochrome P450 345B1 |
| 26073 | 259 | 1.66 | 0.01 | -7.37 | cytochrome P450 CYP349b1 |
| 11451 | 299 | 0.72 | 0.01 | -6.17 | cytochrome P450 CYP49a1 |
| CL3701.  Contig1 | 860 | 4.99 | 0.82 | -2.60 | cytochrome P450 301A1 |
| 9705 | 202 | 7.44 | 1.17 | -2.67 | nicotinic acetylcholine receptor alpha 2 |
| 9791 | 488 | 9.23 | 5.81 | -0.67 | nicotinic acetylcholine receptor alpha 8 |
| 26388 | 254 | 4.22 | 0.01 | -8.72 | nicotinic acetylcholine receptor beta 1 subunit isoform 1 precursor |
| 8062 | 284 | 3.78 | 0.01 | -8.56 | nicotinic acetylcholine receptor a11 subunit splice variant |
| 6968 | 366 | 5.28 | 1.29 | -2.03 | nicotinic acetylcholine receptor alpha1 subunit precursor |
| 25833 | 281 | 2.29 | 0.84 | -1.45 | nicotinic acetylcholine receptor alpha1 subunit precursor |
| 4611 | 305 | 3.52 | 1.55 | -1.18 | nicotinic acetylcholine receptor alpha 8 subunit isoform 1 precursor |
| 22871 | 1605 | 9.89 | 6.03 | 0.71 | carboxylesterase |
| 10857 | 423 | 1747.58 | 863.92 | -1.02 | similar to NADH: ubiquinone dehydrogenase, putative |
| 11068 | 751 | 65.15 | 30.19 | -1.02 | similar to cytochrome P450 CYP6BK17 |
| 1957 | 1223 | 105.97 | 50.61 | -1.05 | cytochrome P450-like protein |
| 10907 | 643 | 221.92 | 105.07 | -1.07 | NADH dehydrogenase |
| 19143 | 564 | 105.00 | 49.00 | -1.09 | cytochrome P450 9Z6 |
| 21415 | 864 | 284.12 | 130.69 | -1.12 | NADH dehydrogenase (ubiquinone) flavoprotein 2 |
| 20293 | 510 | 133.79 | 59.75 | -1.16 | similar to mitochondrial NADH: ubiquinone oxidoreductase ESSS subunit, putative |
| 358 | 761 | 180.18 | 78.84 | -1.19 | NADH dehydrogenase (ubiquinone) Fe-S protein 4 |
| 17553 | 409 | 125.39 | 53.71 | -1.22 | similar to mitochondrial NADH-ubiquinone oxidoreductase AGGG subunit |
| 18776 | 635 | 197.35 | 82.21 | -1.26 | NADH dehydrogenase (ubiquinone) 1 beta subcomplex, 7 |
| 5590 | 292 | 2661.67 | 1096.98 | -1.28 | NADH dehydrogenase subunit 1 (mitochondrion) |
| 1258 | 2192 | 75.47 | 38.15 | -0.98 | peroxidase precursor |
| 23692 | 1359 | 248.37 | 101.34 | -1.29 | NADH dehydrogenase (ubiquinone) 1 alpha subcomplex, 10 |
| 1734 | 1123 | 43.18 | 17.25 | -1.32 | cytochrome P450 9Z4 |
| 1305 | 2487 | 31.49 | 12.63 | -1.32 | similar to ionotropic glutamate receptor subunit ia |
| CL3352.  Contig1 | 543 | 145.42 | 57.42 | -1.34 | NADH dehydrogenase (ubiquinone) Fe-S protein 5, 15kDa (NADH-coenzyme Q reductase) |
| CL3364.  Contig1 | 1370 | 189.36 | 72.42 | -1.39 | similar to NADH-ubiquinone oxidoreductase 39 kda subunit |
| 23286 | 768 | 197.54 | 73.51 | -1.43 | NADH:ubiquinone reductase 23kD subunit |
| CL2781.  Contig1 | 876 | 643.74 | 612.94 | -0.07 | trypsin 3 |
| CL2179.  Contig1 | 1314 | 20.25 | 7.37 | -1.46 | nicotinic acetylcholine receptor alpha 10 subunit precursor |
| 22737 | 950 | 140.13 | 37.54 | -1.47 | similar to Copper chaperone for superoxide dismutase |
| CL910.  Contig2 | 352 | 64.01 | 20.80 | -1.62 | NADH dehydrogenase (ubiquinone) 1 alpha subcomplex, 7, 14.5kDa |
| 7833 | 629 | 125.01 | 40.18 | -1.64 | similar to NADH:ubiquinone dehydrogenase, putative |
| CL1598.  Contig1 | 1761 | 50.32 | 14.89 | -1.76 | Unknown carboxylesterase 2 |
| 19674 | 287 | 84.49 | 22.22 | -1.93 | NADH-ubiquinone oxidoreductase B8 subunit |
| 22329 | 942 | 50.11 | 12.54 | -1.99 | trypsin-like serine proteinase |
| 11378 | 525 | 13.49 | 3.15 | -2.09 | cytochrome P450 307a1-like |
| 13522 | 345 | 16.79 | 2.74 | -2.62 | similar to phosphodiesterase 10A |
| 14701 | 806 | 29.55 | 3.52 | -3.07 | voltage gated chloride channel domain-containing protein |
| 13412 | 648 | 18.54 | 2.19 | -3.08 | Glutathione S-transferase 1 |
| 13523 | 392 | 39.96 | 4.22 | -3.24 | similar to phosphodiesterase 10A |
| CL636.  Contig1 | 645 | 31.27 | 3.29 | -3.25 | cyclic nucleotide phosphodiesterase, putative |
| 24507 | 436 | 23.13 | 1.63 | -3.83 | similar to phosphodiesterase 10A |
| 12643 | 333 | 11.59 | 0.71 | -4.03 | trypsin-like |
| 12135 | 398 | 9.70 | 0.59 | -4.03 | putative trypsin-6-like |
| 1897 | 426 | 26.19 | 0.55 | -5.56 | NADH dehydrogenase (ubiquinone) 1 alpha subcomplex, 7, 14.5kDa |
| 22386 | 1950 | 8.58 | 1.57 | -2.45 | chitinase |
| 1157 | 435 | 2.47 | 16.29 | 2.72 | cuticular protein PxutCPR146, partial |
| 1717 | 1036 | 14.50 | 47.43 | 1.71 | cuticular protein PxutCPR146, partial |
| CL172.  Contig1 | 254 | 22.81 | 0.01 | -11.16 | cuticular protein 43 precursor |
| 17900 | 266 | 13.71 | 0.01 | -10.42 | cuticular protein 16 precursor |
| 18903 | 638 | 829.73 | 0.74 | -10.13 | similar to cuticular protein 6, RR-2 family (AGAP001669-PA) |
| CL2859.  Contig1 | 516 | 14.97 | 0.46 | -5.03 | cuticular protein 28 precursor |
| 654 | 226 | 19.94 | 1.05 | -4.25 | cuticular protein 41 precursor |
| 5102 | 533 | 8.05 | 0.89 | -3.18 | cuticular protein-like precursor |
| 8376 | 667 | 16.73 | 6.02 | -1.47 | similar to cuticular protein 78, RR-1 family (AGAP009876-PA) |
