# Supplementary material for: De Novo Sequencing-Based Transcriptome and Digital Gene Expression Analysis Reveals Insecticide Resistance-Relevant Genes in Propylaea japonica (Thunberg) (Coleoptea: Coccinellidae)
Source: PLoS One. 2014 Jun 24;9(6):e100946. doi: 10.1371/journal.pone.0100946 (PMC4069172; doi:10.1371/journal.pone.0100946)
Supplement: Table S7 — Representatives of putative indirectly related to insecticide resistant genes as predicted by DGE. Limitations of all significantly different expressed genes between R-mid and SUS are based on FDR≤0.001 and the absolute value of log2Ratio≥1. The log2Ratio(R-mid/SUS) indicates the change of gene expression; a positive number means up-regulation and a negative one means down-regulation. (DOC) [file pone.0100946.s013.doc]

Table S7 Representatives of putative indirectly related to insecticide resistant genes as predicted by DGE. Limitations of all signiﬁcantly different expressed genes between R-mid and SUS are based on FDR≤0.001 and the absolute value of log2Ratio≥1. The log2Ratio(R-mid/SUS) indicates the change of gene expression; a positive number means up-regulation and a negative one means down-regulation.

| Gene ID  ( Unigene-) | Length | RPKM  -SUS | RPKM  -R-mid | Log2Ratio (R-mid/SUS) | Annotation |
| --- | --- | --- | --- | --- | --- |
| 9924 | 356 | 9.04 | 0.01 | -9.82 | aminopeptidase N-like |
| 799 | 2612 | 43.29 | 17.73 | -1.29 | aminopeptidase-like protein |
| 12 | 912 | 36.47 | 15.54 | -1.23 | aminopeptidase N-like protein |
| 4541 | 264 | 1.63 | 16.11 | 3.31 | similar to actin binding |
| 10723 | 479 | 89.15 | 309.70 | 1.79 | similar to antennal-enriched UDP-glycosyltransferase |
| CL52.Contig1 | 1591 | 40.87 | 121.60 | 1.57 | similar to antennal-enriched UDP-glycosyltransferase |
| 24389 | 374 | 1.60 | 0.01 | 10.74 | heat shock protein 90 |
| 6793 | 431 | 0.01 | 7.13 | 9.48 | heat shock protein 70, putative |
| 8013 | 211 | 0.01 | 3.36 | 8.39 | heat shock protein 90 |
| 4776 | 244 | 0.01 | 0.97 | 6.59 | heat shock protein 90kDa beta |
| 24714 | 335 | 0.64 | 11.28 | 4.14 | heat shock protein 90, partial |
| 3804 | 1161 | 0.55 | 6.71 | 3.59 | heat shock protein 70 precursor |
| 19311 | 882 | 678.29 | 3597.18 | 2.41 | similar to small heat shock protein 21 |
| 25764 | 213 | 2.01 | 4.44 | 1.14 | heat shock protein 90 |
| CL133.Contig4 | 242 | 5.32 | 0.01 | -9.05 | similar to small heat shock protein 21 |
| CL133.Contig2 | 287 | 42.62 | 4.12 | -3.37 | similar to small heat shock protein 21 |
| CL2876.Contig1 | 684 | 26.35 | 3.45 | -2.93 | heat shock protein 70 |
| CL133.Contig1 | 394 | 4.36 | 0.60 | -2.86 | similar to small heat shock protein 21 |
| 21217 | 1027 | 20.27 | 3.46 | -2.46 | heat shock protein TC005094 |
| 13971 | 251 | 10.26 | 1.88 | -2.45 | heat shock protein 70 |
| 6214 | 227 | 5.67 | 1.04 | -2.45 | similar to heat shock protein 40 |
| CL3984.Contig1 | 282 | 11.41 | 2.51 | -2.18 | heat shock protein 70 |
| CL133.Contig3 | 728 | 19.75 | 4.54 | -2.12 | similar to small heat shock protein 21 |
| CL2464.Contig2 | 997 | 112.78 | 36.02 | -1.64 | similar to heat shock protein |
| 8243 | 456 | 27.29 | 8.81 | -1.63 | similar to small heat shock protein 21 |
| 22759 | 818 | 453.03 | 156.81 | -1.53 | similar to heat shock protein 1 |
| 12101 | 424 | 11.64 | 36.77 | 1.66 | similar to ATP-binding cassette transporter |
| 16080 | 385 | 22.85 | 67.49 | 1.56 | similar to ATP-binding cassette transporter |
| 12322 | 369 | 22.68 | 52.49 | 1.21 | similar to ATP-binding cassette transporter |
| 16079 | 326 | 39.49 | 90.58 | 1.20 | similar to ATP-binding cassette transporter |
| 7143 | 223 | 0.01 | 2.12 | 7.73 | vacuolar ATP synthase, catalytic subunit a |
| 26296 | 273 | 0.01 | 0.87 | 6.44 | putative ATP synthase-like protein |
| 506 | 380 | 25.41 | 0.01 | -11.31 | ATP synthase beta subunit |
| CL1562.Contig1 | 357 | 13.22 | 0.01 | -10.37 | ATP synthase subunit beta, mitochondrial-like |
| 10557 | 219 | 9.80 | 0.01 | -9.94 | hypothetical protein |
| 13557 | 282 | 7.61 | 0.01 | -9.57 | ATP synthase subunit s, mitochondrial |
| 12239 | 313 | 17.82 | 0.75 | -4.56 | ATP synthase, H+ transporting, mitochondrial F0 complex, subunit s |
| 6995 | 422 | 15.76 | 3.36 | -2.23 | ATP synthase B chain, mitochondrial |
| 24463 | 337 | 1.91 | 0.70 | -1.44 | ATP synthase c-subunit-like |
| CL3249.Contig1 | 488 | 451.15 | 166.52 | -1.44 | similar to H+ transporting ATP synthase subunit e |
| 19323 | 619 | 614.62 | 275.53 | -1.16 | similar to ATP synthase delta chain, mitochondrial |
| 19357 | 622 | 1529.66 | 686.64 | -1.16 | similar to mitochondrial F0 ATP synthase D chain, putative |
| CL2390.Contig1 | 1842 | 2542.58 | 1214.97 | -1.07 | ATP synthase subunit beta, mitochondrial |
| 1518 | 326 | 959.03 | 468.82 | -1.06 | similar to mitochondrial ATP synthase coupling factor 6 |
| 20025 | 525 | 1316.09 | 648.38 | -1.02 | similar to hydrogen- transporting ATP synthase, G-subunit, putative |
